# Supplementary material for: Development and performance evaluation of a solenoid valve assisted low-cost ventilator on gas exchange and respiratory mechanics in a porcine model
Source: PLoS One. 2024 May 16;19(5):e0303443. doi: 10.1371/journal.pone.0303443 (PMC11098403; doi:10.1371/journal.pone.0303443)
Supplement: Attachment — Submitted filename: LCV Response Letter.docx [file pone.0303443.s001.docx]

**Response to Referee and Editor Comments on the Paper:**

**“To Test the Performance of an Open-Source, Low-Cost Ventilator in a Porcine Model”**

**PLOS One Journal**

**PONE-D-23-12462**

Response to Editor’s Comments:

For this revision, the Editor recommends that we consider the suggestions by the reviewer. This response note forms the main part of the requested accompanying letter. We also note here that we have been able to accommodate all the suggestions proposed by both reviewers. Below is the summary of the suggestions and our responses to them.

Reviewer #1

1. I am a little unclear what the author's expectations were for this article. They claim an open-source design, one that is not thoroughly given or expanded upon. Additionally, the timeline of this topic is misaligned with the reality of current health states. The federal government has removed the state of emergency over COVID-19 and there have been several papers on this or a similar topic. Additionally, proposing an "open source" medical instrument like a ventilator is a very precarious position to take as the amount of safety concerns was also something brought up when teams were in the midst of the pandemic working on rapid response ventilators. There are several other papers exactly like this paper, including the ones cited and published in this journal that did porcine modeling as well. The novelty of this paper is therefore in question and the value that it adds to the journal and to the field I think is questionable. The reference list is woefully short for this kind of important paper and the depth of analysis, risk analysis, part sourcing is lacking. The animal study that was claimed was not detailed with any thoroughness or explanation. This paper is far too short and underdeveloped for the important concepts introduced. There are many other papers, including ones the authors cite that cover this work in better detail, in PLOS ONE no less. Therefore, I suggest the editor reject this submission.

**Response: Appreciate the comments and agree with the consensus on the numerous reports for low-cost ventilators that have been produced during the COVID pandemic. However, we would like to provide reasoning and support on why our LCV study is unique compared to others that have been previously published. In a PubMed search for “low-cost ventilator”, there were 14 relevant published papers in the first 150 searches. The table below demonstrates the clear differences between our study and the other 14 publications:**

|  | **Current Study** | **14 LCV publications** |
| --- | --- | --- |
| **Type of ventilator** | **Solenoid valve** | **2 of 14** |
| **Use of animal model** | **Yes** | **No** |
| **Comparison to control ventilator** | **Yes** | **No** |
| **Comparative demonstration using physiological data (i.e. arterial blood gas)** | **Yes** | **No** |

**We believe these key differences are novel and adds to the scarcity of the current literature available. Moreover, have been limitations for other publications.**

**We appreciate that we have not clearly presented these differences in our manuscript. In this revision, we have reflected these differences and provided additional references to support the discussions and our findings as well. The title, introduction and discussion paragraphs have been revised to highlight what stands out in our design in addition to emphasizing that we have physiological gas exchange data that has not been reported previously for LCV during the COVID pandemic.**

Reviewer #2

1. There is no description of the anesthetic protocol or management for the porcine model. It is beneficial for the reader to understand what type of anesthetics were used for this study and what effect the anesthetics could have on the parameters evaluated.

**Response: Agree. Added in the methods section.**

2. Please make sure all acronyms are spelled out during their initial use.

**Response: Agree. Our manuscript has been revised.**

3. For the lung simulator testing, the duration of testing for each condition is unclear.

**Response: Agree. Our manuscript has been revised.**

4. Table 3 - Ti and Te for control ventilator were not provided in the chart. Are those values available? The legend of table 3 also appears cut off (no description of what PO2 is)

**Response: The Ti and Te chosen for this study resulted from calibration using our lung simulator. This provided us with the tidal volumes demonstrated in the porcine model. Legend for Table 3 has been corrected.**

5. Recommended citation: King, W. P., Amos, J., Azer, M., Baker, D., Bashir, R., Best, C., ... & Wooldridge, A. R. (2020). Emergency ventilator for COVID-19. PLoS One, 15(12), e0244963.

**Response: Agree. Citation has been added.**
