# Supplementary material for: Development and performance evaluation of a solenoid valve assisted low-cost ventilator on gas exchange and respiratory mechanics in a porcine model
Source: PLoS One. 2024 May 16;19(5):e0303443. doi: 10.1371/journal.pone.0303443 (PMC11098403; doi:10.1371/journal.pone.0303443)
Supplement: Attachment — Submitted filename: LCV Response Letter2.docx [file pone.0303443.s002.docx]

**Response to Referee and Editor Comments on the Paper:**

**“To Test the Performance of an Open-Source, Low-Cost Ventilator in a Porcine Model”**

**PLOS One Journal**

**PONE-D-23-12462**

Response to Editor’s Comments:

For this revision, the Editor recommends that we consider the suggestions by the reviewer. This response note forms the main part of the requested accompanying letter. We also note here that we have been able to accommodate all the suggestions proposed by both reviewers. Below is the summary of the suggestions and our responses to them.

Reviewer #3

1. Write the applications in the introduction section.

Response: The major application for this LCV device is under emergency use ventilators which is well explained in the introduction section. We did revise the manuscript to other potential uses for the LCV as well. This would include underserved medical areas and potential applications for the LCV.

2. Add quantitative results in the abstract.
Response: Done. Abstract has been revised

3. Provide references to support your results.

Response: Done. Please refer to references#11-15. Second paragraph of the discussion section has been revised to make this point. Reference#12 is a new study that was added to enhance the support

4. The results section needed to be improved as it seems to be a laboratory report rather than a scientific investigation.

Response: revised

5. Reconstructed the conclusion part.

Response: Done. Conclusion section separated and revised.

Roy Cho MD, MHA

University of Minnesota
